# Awareness of Carpal Tunnel Syndrome Among the Adult Population in Northern Saudi Arabia

**DOI:** 10.7759/cureus.52100

**Published:** 2024-01-11

**Authors:** Mohamed M Abd El Mawgod, Khalid M Alhazmi, Adel S Alanazi, Salem M Alenezi, Wael A Alanazi

**Affiliations:** 1 Public Health and Community Medicine, Faculty of Medicine, Al-Azhar University, Assiut, EGY; 2 Family and Community Medicine, College of Medicine, Northern Border University, Arar, SAU; 3 College of Medicine, Northern Border University, Arar, SAU

**Keywords:** risk factors, signs, symptoms, awareness, carpal tunnel syndrome

## Abstract

Background

Carpal tunnel syndrome (CTS) is the most prevalent entrapment neuropathy affecting the upper limb. It is recognized as a complex condition that is attributed to both non-medical and medical risk factors. Lack of awareness leads to delays in seeking advice, diagnosis, and treatment.

Objective

To determine the awareness of CTS, its associated symptoms, signs, and risk factors among the adult population.

Subjects and methods

A cross-sectional study design was carried out among the adult population in Arar city, Northern Saudi Arabia.

Results

In total, 338 respondents participated in this study. More than one-third (40.8%) mentioned that median nerve entrapment is a cause of CTS. The most commonly cited risk factor by the respondents was engaging in physical tasks such as using a computer (53%). Additionally, 60% of participants agreed that symptoms of CTS include tingling and numbness in the thumb, index, and middle fingers.

Conclusion

The findings of the study indicated a lack of adequate community awareness about CTS among the studied population.

## Introduction

Carpal tunnel syndrome (CTS) is a medical condition that is indeed caused by the compression of the median nerve as it passes through the carpal tunnel, a narrow passageway located in the wrist [1]. In Saudi Arabia, the prevalence of CTS varied from 14% to 30.5%, but for individuals with diabetes, it reached 10%-56.1% [2, 3]. It is particularly prevalent in people with certain medical conditions or those in predisposed professions [4]. There are numerous causes of CTS, including rheumatoid arthritis, diabetes mellitus, obesity, work requiring manual tasks [5], and repetitive motions of the hands and wrists [6]. CTS leads to dysfunctions as a result of compression [7]. The symptoms of CTS include pain, paresthesia, weakness and numbness in the thumb's opposition and abduction, and difficulties gripping objects [8]. It interferes with tasks that are necessary for daily living, like cooking, cleaning teeth, and holding objects with one hand [9]. Pain, numbness, paresthesia, weakness, and temperature fluctuations in the affected limb are the most common symptoms of CTS. Many individuals have stated that their symptoms worsen at night or are brought on by specific activities [10, 11]. Additionally, it has been noted that 73% of CTS cases exhibit bilateral symptoms, typically affecting the dominant hand first, even when the manifestations do not occur simultaneously [12]. CTS affects the quality of life and impairs hand function, mainly when patients express pain [8]. According to a study conducted in Hail, Saudi Arabia, CTS can interfere with daily tasks and lower one's quality of life [5]. Albaker AB et al. (Saudi Arabia) reported that 55.9% of people were aware of CTS, with 38.3% being aware of its associated pain. Among the participants, 37.3% reported experiencing tingling or numbness in their thumbs, 27.8% believed that CTS could cause thumb weakness, and 35.2% observed decreased hand grip strength [13]. In a Brazilian study, participants mentioned experiencing pain and difficulties with tasks requiring delicate hand motions. This affected activities at home, in the workplace, and during leisure time [14]. Few studies have been conducted in Saudi Arabia to assess the awareness of CTS, its symptoms, signs, and risk factors. To our knowledge, no studies have been specifically conducted in the northern region. The current study aims to determine the awareness of CTS and its associated symptoms, signs, and risk factors among the adult population in Arar city, Northern Saudi Arabia.

## Materials and methods

Study design and setting

A cross-sectional study design was carried out from March 1 to September 30, 2023, among an adult population in Arar city, Northern Saudi Arabia.

Inclusion and exclusion criteria

Subjects who were willing to participate and were older than 18 were included in the study; those who declined to participate or were younger than 18 were not.

Research ethics

The Local Committee of Bioethics at Northern Border University approved the research (HAP-09-A-043) with a decision no. 35-44-H, dated March 29, 2023. With informed consent, participation was voluntary. Confidentiality was ensured, and we kept the collected data safe.

Methodology

A structured questionnaire was developed by searching pertinent literature and revised by two physicians (family and orthopedic). In order to resolve any ambiguities, the questionnaire was piloted on 20 subjects (their results were not included in the analysis) before it was actually administered. The questionnaire included questions related to socio-demographic backgrounds, risk factors, symptoms, protective factors, and the effects of CTS.

The sample size was calculated by using the following formula: N = Z2(p)x(1-p)/d2, where N = sample size, Z = confidence level (1.96), P = the expected prevalence of awareness (30%) from the previous study [15], and d = precision (0.05). The expected sample size was 323, and we completed the sample at 338.

Following ethical permission, a survey was carried out via social media platforms (WhatsApp, Telegram). After discussing the research objective, consent was obtained from the willing participants for enrollment in the study.

Statistical analysis

The collected data was analyzed using SPSS version 22. Frequency and percentage were used to present qualitative data, and mean and SD were used to present quantitative data.

## Results

In total, 338 respondents participated in this study; their mean age was 29.5±9.8, with slightly more women than men (52.4%), around 50% were married, 50% had a university degree, and more than one-third unemployed (37%) (Table 1).

**Table 1:** Sociodemographic backgrounds of the study participants.

Item	No. of respondents (338)	%
Sex		
Male	161	47.6
Female	177	52.4
Marital status		
Single	147	43.5
Married	168	49.7
Divorced/Widowed	23	6.8
Educational level		
Illiterate	15	4.4
Primary school	11	3.3
Intermediate school	18	5.3
High school	122	36.1
University	172	50.9
Occupation		
Unemployed	125	37.0
Student	84	24.8
Employee	123	36.4
Retired	6	1.8

Table 2 demonstrates the participants' awareness of risk factors for CTS. More than one-third of them mentioned that median nerve entrapment is a cause (40.8%). The most commonly cited risk factor was engaging in physical tasks such as using a computer (53%), followed by trauma (47%), arthritis (38.2%), rheumatoid arthritis (RA) (37.6%), wrist fractures or dislocations (33.7%), bone tumors (29.9%), hypothyroidism (25.7%), and diabetes mellitus (25.4%).

**Table 2:** Participants' awareness of CTS risk factors. CTS: Carpal Tunnel Syndrome.

Item	No. of respondents (338)	%
CTS is caused by median nerve compression		
Yes	138	40.8
No	84	24.9
I don't know	116	34.3
Repetitive activities of the hand and wrist can cause CTS		
Yes	179	53.0
No	50	14.8
I don't know	109	32.2
Trauma can cause CTS		
Yes	159	47.0
No	52	15.4
I don't know	127	37.6
Arthritis can cause CTS		
Yes	129	38.2
No	73	21.6
I don't know	136	40.2
Rheumatoid arthritis (RA) has a relation with CTS		
Yes	127	37.6
No	58	17.2
I don't know	153	45.2
Wrist fractures or dislocation can cause CTS		
Yes	114	33.7
No	79	23.4
I don't know	145	42.9
Bone tumors can cause CTS		
Yes	101	29.9
No	80	23.7
I don't know	157	46.4
Hypothyroidism has a relation with CTS		
Yes	87	25.7
No	81	24.0
I don't know	170	50.3
Diabetes mellitus has a relation with CTS		
Yes	86	25.4
No	81	24.0
I don't know	171	50.6

Table 3 illustrates the participants' awareness of protective factors for CTS. Slightly fewer than forty percent mentioned that preventing CTS involves keeping the wrist straight while at rest. More than one-third (37%) stated that avoiding falls is important, followed by 34.3% who emphasized keeping warm. Additionally, one-third of the participants (33.1%) reported that wearing a splint while sleeping is beneficial.

**Table 3:** Participants' awareness of CTS protective factors. CTS: Carpal Tunnel Syndrome.

Item	No. of respondents (338)	%
Keeping the wrist straight while at rest prevents CTS		
Yes	134	39.6
No	67	19.8
I don't know	137	40.6
Avoiding falls or direct impact prevents CTS		
Yes	125	37.0
No	77	22.8
I don't know	136	40.2
Keeping the wrist warm prevents CTS		
Yes	116	34.3
No	64	18.9
I don't know	158	46.8
Wear a splint while sleeping prevents CTS		
Yes	112	33.1
No	79	23.4
I don't know	147	43.5

Table 4 displays the participants' awareness of the symptoms of CTS. Sixty percent of participants agreed that tingling and numbness in the thumb, index, and middle fingers are symptoms of CTS. More than half (56.4%) concurred that CTS causes pain in the wrist. Slightly below half (48%) agreed that CTS leads to weakening in the thumb muscles, and somewhat less than 60% believed that the intensity of pain changes with wrist movement.

**Table 4:** Participants' symptoms awareness of CTS. CTS: Carpal Tunnel Syndrome.

Item	No. of respondents (338)	%
CTS causes numbness and tingling in the middle, index, and thumb fingers		
Strongly disagree	7	2.1
Disagree	11	3.3
Not decided	117	34.6
Agree	124	36.7
Strongly agree	79	23.3
CTS causes pain in wrist		
Strongly disagree	8	2.4
Disagree	11	3.3
Not decided	128	37.9
Agree	110	32.4
Strongly agree	81	24.0
CTS causes weakness, affecting the thumb muscles		
Strongly disagree	7	2.1
Disagree	27	8.0
Not decided	142	42.0
Agree	107	31.7
Strongly agree	55	16.2
Patients with CTS experience changing pain levels as they move their wrist		
Strongly disagree	8	2.4
Disagree	14	4.1
Not decided	124	36.7
Agree	120	35.5
Strongly agree	72	21.3

Table 5 shows that more than half of the participants agreed that CTS reduces hand grip strength (50.5%) and that it typically starts slowly and lasts throughout the night (51.8%). Slightly less than half of the respondents agreed that CTS causes wasting of the hand muscles (46.4%) and that CTS sufferers experience discomfort at night and in the morning (47.6%). Additionally, just below one-third agreed that CTS can occur in both hands.

**Table 5:** Participants' symptoms awareness of CTS. CTS: Carpal Tunnel Syndrome.

Item	No. of respondents (338)	%
Carpal tunnel syndrome decreases overall hand grip		
Strongly disagree	10	3.0
Disagree	27	8.0
Not decided	130	38.5
Agree	99	29.2
Strongly agree	72	21.3
Symptoms of CTS usually begin slowly and overnight		
Strongly disagree	8	2.4
Disagree	19	5.6
Not decided	136	40.2
Agree	119	35.2
Strongly agree	56	16.6
CTS causes wasting muscles of hand		
Strongly disagree	5	1.5
Disagree	26	7.7
Not decided	150	44.4
Agree	101	29.8
Strongly agree	56	16.6
CTS sufferers have discomfort at night and in the morning		
Strongly disagree	8	2.4
Disagree	15	4.4
Not decided	154	45.6
Agree	103	30.5
Strongly agree	58	17.1
Carpal tunnel syndrome affects both hands		
Strongly disagree	27	8.0
Disagree	55	16.3
Not decided	159	47.0
Agree	62	18.3
Strongly agree	35	10.4

Table 6 shows the participants' awareness of the effects of CTS. A little over half of them (53.8%) agreed that CTS impacts patients' ability to perform their jobs, while slightly under half (48.2%) agreed that it affects patients' sleep. Around 40% concurred that the disease affects social life. A total of 12.4% of the participants believed that they had CTS. Less than 30% stated that the disease can be treated using steroids (29%), oral analgesics (26.9%), and splints (26.6%).

**Table 6:** Participants' knowledge of the effects of CTS. CTS: Carpal Tunnel Syndrome.

Item	No. of respondents (338)	%
CTS affects patients' job performance		
Strongly disagree	11	3.3
Disagree	12	3.6
Not decided	133	39.3
Agree	111	32.8
Strongly agree	71	21.0
CTS affects patients' sleep		
Strongly disagree	6	1.8
Disagree	27	8.0
Not decided	142	42.0
Agree	101	29.9
Strongly agree	62	18.3
CTS affects social life		
Strongly disagree	49	14.4
Disagree	28	8.3
Not decided	123	36.4
Agree	81	24.0
Strongly agree	57	16.9
Do you think you suffer from CTS?		
Yes	42	12.4
No	224	66.3
I do not know	72	21.3
Oral analgesics can treat CTS		
Yes	91	26.9
No	105	31.1
I do not know	142	42.0
NSAIDs can treat CTS		
Yes	106	31.4
No	71	21.0
I do not know	161	47.6
Steroid injections can treat CTS		
Yes	98	29.0
No	66	19.5
I do not know	174	51.5
Splints can treat CTS		
Yes	90	26.6
No	92	27.2
I do not know	156	46.2

## Discussion

In the current study, slightly more than 40% of participants believed that CTS was caused by median nerve compression. Comparable research in the Al-Jouf region of Saudi Arabia, conducted by Alqunai MS in 2021, revealed that slightly more than 25% of participants had heard of CTS and understood that it is caused by median nerve entrapment [16]. Similarly, a study by Kandhan T et al. in India found that 28.6% of participants were aware of CTS [17]. Additionally, a majority of respondents in a nationwide study conducted in Rome recognized that CTS is caused by compression of the median nerve [18]. Regarding the participants' awareness of Carpal Tunnel Syndrome (CTS) risk factors, repetitive motions of the hands and wrists were cited most frequently (53%), while diabetes mellitus was stated less frequently (25.4%). This is in line with a comparable study conducted in Saudi Arabia [19], where 52.7% of the respondents reported repetitive hand and wrist motions, 37.3% cited wrist fracture or dislocation, 56.3% arthritis, and 41.6% bone tumors as risk factors for CTS. Additionally, Alqunai MS (2021) in Saudi Arabia discovered that about 30% of participants stated that engaging in repetitive hand and wrist activity could increase the chance of acquiring CTS [16]. 

Alyousef YM et al. in Saudi Arabia reported that one-third (33.8%) of their study participants believe that trauma is a primary cause of CTS, while 29.1% mentioned that repetitive physical activities such as computer use and typing are the reason. Additionally, 21.4% indicated wrist fracture or dislocation, 9.5% arthritis, and 6.2% bone tumors as contributing factors [15].

According to case-control studies conducted in Saudi Arabia [20] and China [21], using a mobile phone with repetitive hand movements was found to increase the chance of acquiring CTS. On the other hand, Scalise V et al. in Rome, Italy, discovered that a significant portion of participants (88.2%, or 448 out of 508) accurately identified diabetes mellitus as a risk factor for CTS. Additionally, 11.6% (59 out of 508) cited hormonal factors, and 58.7% considered the use of computers as contributing to the risk of developing CTS [18].

In terms of awareness of protective factors for CTS, the study shows that keeping a straight wrist at rest is most frequent, followed by avoiding falls, keeping the wrist and hand warm, and using a splint while sleeping. On the other hand, the comparable study conducted in western Saudi Arabia revealed different percentages of participants mentioning protective factors like avoiding direct trauma (25.6%), keeping wrists straight at rest (23.3%), using splints while sleeping (14.3%), and staying warm [22]. Regarding symptom awareness among the study sample, 60% agreed that numbness and tingling in the middle, index, and thumb fingers are symptoms of CTS. Slightly less than 60% mentioned wrist pain, just below half stated weakness of the thumb muscles, and more than half cited changes in pain intensity as indicators of CTS. In a similar Italian study, Scalise V et al. found that 81.7% of participants correctly identified the characteristic symptoms of CTS as tingling and numbness in the first three fingers of the hand. Additionally, 2.6% proposed that joint limitation due to a loss of hypothenar muscular power was a symptom. A correct response regarding the hypotrophy of the thenar eminence was demonstrated by 74.8% of participants [18]. The current study reveals that over 50% of respondents agree that CTS can lead to a reduction in hand grip strength, typically beginning slowly overnight. Additionally, just under one-third of the participants concur that CTS can manifest in both hands. This is in line with a similar survey in Saudi Arabia, where more than half of the respondents (57%) mentioned a decrease in hand grip strength, and 40% noted that the condition often starts at night [19]. In the Alqunai MS study, also conducted in Saudi Arabia, 23.3% of participants reported that CTS manifests in both hands, 32% reported hand weakness, and one-fifth mentioned that CTS starts slowly overnight [16]. According to the survey results, slightly more than half of the respondents believe that Carpal Tunnel Syndrome (CTS) influences a patient's ability to perform their job effectively. Just under half of the participants mentioned that CTS affects the quality of a patient's sleep, while approximately 40% agreed that it impacts a patient's social life. In a similar study conducted in Saudi Arabia, 51.1% of respondents thought that CTS could affect work performance, 28.4% believed it influenced sleep, and 20.5% recognized its impact on social life [22].

Study limitations

The study was conducted using a non-probability sample in Arar city, which limits its generalizability. Additionally, the absence of a standardized questionnaire presents another constraint. However, given the exploratory nature of this work, it provides valuable insights and ideas for further research.

## Conclusions

The current study presents strong evidence that adult residents of Arar city, Northern Saudi Arabia, possess a relatively low level of awareness concerning the risk factors, protective factors, symptoms, and effects of CTS. These findings underscore the necessity of taking prompt and meaningful action to address this issue. To enhance awareness, educational campaigns about the risk factors and symptoms of CTS should be conducted at various public and private locations. Such initiatives will aid in encouraging patients to seek medical attention early and contribute to the improved management of the condition.
